# Serum Aldosterone Is Related to Left Ventricular Geometry and Function in Young Adults with Never-Treated Primary Hypertension

**DOI:** 10.3390/jcm8071045

**Published:** 2019-07-17

**Authors:** Seong-Mi Park, Mi-Na Kim, Sua Kim, Wan-Joo Shim

**Affiliations:** 1 Division of Cardiology, Korea University Anam Hospital, Korea University College of Medicine, 73, Inchon-ro, Seongbuk-gu, Seoul 02841, Korea; 2 Division of Intensive Care Medicine, Korea University College of Medicine, 123 Jeokgeum-ro, Danwon-gu, Ansan 15355, Korea

**Keywords:** young, hypertension, aldosterone, hypertrophy, cardiac function

## Abstract

Background: Although aldosterone has been demonstrated to induce left ventricular (LV) hypertrophy not only in primary aldosteronism but also in primary hypertension (HT), it can be affected by multiple factors, including age, and the effect of aldosterone on LV function is controversial. This study was to investigate the relationship of aldosterone to changes in LV geometry and function in young adults with never-treated HT. Methods: Seventy-five consecutive patients (age, 29.8 ± 6.3 years) with never-treated HT and 45 normal controls were enrolled. Echocardiographic values and LV global longitudinal strain (LVGLS) were obtained. Serum aldosterone concentration (SAC) and serum procollagen type III amino-terminal peptide (PIIINP) level were obtained in HT patients. Results: HT patients had higher LV mass index, higher relative wall thickness (RWT), and worse LV function than normal controls. LVGLS and e’ velocity were worse in HT patients with normal geometry than in normal controls. SAC was well correlated with LV mass index, RWT, e’ velocity, LVGLS, and PIIINP (all *p* < 0.05). LV geometry pattern was most related to SAC among clinical parameters (*p* = 0.019). LVGLS was most related to LV geometry and diastolic blood pressure. In contrast, e’ velocity was most related to PIIINP. Conclusion: Our findings may indicate that in young patients with never-treated HT, aldosterone significantly contributes to changes in LV geometry and functional impairment through its pro-hypertrophic and myocardial fibrosis effects beyond blood pressure.

## 1. Introduction

Elevation in blood pressure (BP) increases the afterload and left ventricular (LV) wall stress, resulting in subendocardial damage and myocardial fibrosis, which then lead to LV remodeling with increased LV mass in systemic arterial hypertension (HT). Previous studies have shown that increased LV mass, known as LV hypertrophy (LVH), increases cardiovascular disease risk and is an independent predictor of cardiovascular events [1,2,3]. Many factors beyond BP level contribute to the HT-related changes in LV remodeling, including excess plasma aldosterone levels, which have been reported to increase myocardial mass and collagen content in HT patients [4,5].

Aldosterone excess in primary aldosteronism has been associated with higher risks of HT, kidney damage, and LVH. Recently, it has been shown that circulating aldosterone levels, even within the physiological range, are also related to an increased risk of cardiovascular mortality, fatal stroke, and sudden cardiac death [6]. Aldosterone has been demonstrated to influence LV remodeling independent of its impact on systemic BP [7].

However, the renin–angiotensin–aldosterone system (RAAS) and aldosterone as the end product can be affected by multiple factors, including antihypertensive agents, comorbidities, and aging [8,9,10]. Although young adults with primary HT may have a relatively short duration of high BP, some patients show significant LVH and functional impairment. We sought to determine whether elevated serum aldosterone level is one of the mechanisms of such remodeling. There are limited data about the relationship of RAAS to the changes in LV mass, wall thickness, and function in young adults with never-treated HT. Moreover, the effect of aldosterone on LV systolic and diastolic function is controversial.

Therefore, this study aims to evaluate the relationship of aldosterone to LV geometry, function, and myocardial fibrosis in these patients.

## 2. Methods

### 2.1. Study Subjects

Seventy-five consecutive young patients (age ≤ 40 years) diagnosed with primary arterial HT and with no history of use of anti-HT medications were enrolled. The exclusion criteria were a history of coronary artery disease, diabetes, LV ejection fraction (LVEF) <50%, any valvular or congenital heart disease, atrial fibrillation, and renal dysfunction (plasma creatinine >1.5 mg/dL). Forty-five age- and sex-matched normal healthy subjects were enrolled as controls. These subjects were selected from patients who underwent echocardiography for noncardiac causes and from volunteers, through frequency matching. All subjects had no medical history or specific medication. Based on the 7th Report of the Joint National Committee on Prevention, Detection Evaluation, and Treatment of High Blood Pressure, the HT criteria were systolic BP (SBP) ≥140 mmHg and/or diastolic BP (DBP) ≥90 mmHg [11]. BP recordings were obtained according to the standard technique with the subject in a sitting position. Measurements were performed 3 times with rest intervals of at least 5 min, and then the values were averaged. Twenty-four-hour ambulatory BP monitoring was performed in the nondominant arm by using a validated oscillometric device (SpaceLabs 90207 monitor; SpaceLabs Inc., Redmond, WA, USA). We defined 24 h HT as a 24 h ambulatory BP of at least 130 mmHg systolic or at least 80 mmHg diastolic. Any secondary causes of HT were excluded in all patients according to established guidelines [8]. We excluded 2 patients who had an aldosterone-to-renin ratio (ARR) of at least 20 in the presence of a serum aldosterone concentration (SAC) of at least 15 ng/dL (primary aldosteronism) [12].

All measurements, including BP, echocardiographic study, and blood tests were performed before starting antihypertensive medications. All subjects provided informed consent, and study approval was obtained from the Institutional Review Board of the Korea University College of Medicine.

### 2.2. Conventional Echocardiography

A commercially available echocardiography system (Vivid 7; GE Medical Systems, Horten, Norway) with a 3.5 MHz phased-array transducer was used for conventional 2-dimensional (2D) echocardiography imaging. Echocardiography was performed in accordance with the standards of the American Society of Echocardiography (ASE) [13].

Mitral inflow velocity was obtained using pulsed-wave Doppler echocardiography with the sample volume between mitral leaflet tips during early diastole (E), and its deceleration time (DT) was measured. Early diastolic septal mitral annular velocities were obtained from the pulse wave velocity of spectral tissue Doppler imaging and averaged (e’). The E/e’ was also calculated. LV mass index (LVMI) was calculated from end-diastolic M-mode or 2D-guided measurements of the ventricular septum, LV end-diastolic diameter (LVEDD), LV end-systolic diameter (LVESD), and posterior wall thickness (PWT), according to the method of Devereux et al. [14].

LV relative wall thickness (RWT) and systolic wall stress were calculated as follows:

RWT = 2 × (diastolic PWT ÷ LVEDD)

Systolic wall stress = 0.334 × SBP × LVESD ÷ (systolic PWT × (1+systolic PWT/LVESD))

LVH was defined as an LVMI exceeding 115 g/m^2^ in male patients and 95 g/m^2^ in female patients, respectively, according to the 2015 ASE guideline, and the cutoff point for abnormal RWT was 0.44 [13]. Patterns of LV geometry were defined according to the European Society of Hypertension/European Society of Cardiology and ASE guidelines [11,13], as follows: normal geometry (normal LVMI combined with RWT <0.44), LV concentric remodeling (normal LVMI combined with RWT ≥0.44), eccentric LVH (increased LVMI and RWT <0.44), and concentric LVH (increased LVMI and RWT ≥0.44). All parameters were measured 3 times, and the results were averaged.

### 2.3. Two-Dimensional Speckle-Tracking Imaging

Second-harmonic B-mode images with a frame rate of 77 ± 6 frames/s (range, 63–99 frames/s) were obtained for offline analysis. According to the ASE guidelines [15,16], apical 4- and 2-chamber, and long-axis views were used for longitudinal strain measurement. Echocardiographic recordings were analyzed using dedicated software (EchoPAC-PC, version 10.0; GE Medical Systems, Horten, Norway), and myocardial motion was automatically tracked in each imaging view. A region of interest was manually adjusted to include the entire myocardial thickness, with care taken to avoid including the pericardium. The software then selected stable speckles within the myocardium and tracked these speckles frame-by-frame throughout the cardiac cycle. An 18-segment long-axis LV model was automatically obtained from 3 apical views. To determine the phases of the cardiac cycle, the timings of aortic and mitral valve opening and closure were assessed using pulsed-wave Doppler recordings of aortic and transmitral flow. LV global longitudinal strain (LVGLS, %) was calculated as the mean longitudinal peak negative strain from 18 apical segments during a cardiac cycle (Supplementary Materials File S1). Cardiac cycles with extrasystolic beats, post-extrasystolic beats, or any rhythm disturbances were excluded. Appropriate LVGLS was obtained in all subjects. The inter- and intra-observer reproducibilities were obtained from 15 randomly selected subjects. The inter-observer variability was 5.0%, and the intra-observer variability was 2.5%.

### 2.4. Laboratory Measurements

Venous blood was collected in the morning after a 12 h fast with the patients in the sitting position after an approximately 30 min rest. Plasma was immediately separated and frozen at −80 °C until the assay was performed. The SAC was measured in EDTA plasma (Coat-A-Count Aldosterone; Siemens Healthcare Diagnostics, Los Angeles, CA, USA). The plasma renin activity (PRA) was determined with the radioimmunoassay method and Immunotech kit (Beckman Coulter, Immunotech, Prague, Czech Republic). ARR was calculated as SAC divided by PRA.

The serum procollagen type III amino-terminal peptide (PIIINP) level was measured in 45 of the 75 patients who agreed to undergo blood sampling for this test. Blood samples were centrifuged immediately after sampling, and the separated serum was stored at –40 °C for subsequent analysis. Serum PIIINP levels were measured using a specific immunoradiometric assay (CIS Bio International, Mitsubishi, Japan).

To estimate the 24 h urinary sodium excretion from spot urine samples, we used Tanaka’s prediction model [17]. The estimated glomerular filtrate rate was calculated using the Chronic Kidney Disease Epidemiology Collaboration equation [18].

### 2.5. Statistics

Values were presented as mean ± standard deviation or as numbers and percentages. Comparisons between normal controls and HT patients were made using the Student’s *t*-test for continuous variables. Pearson correlation analysis was used to analyze the correlations between SAC, PRA, ARR, and echocardiographic variables of LV structure and function. The differences in LV geometries in HT patients were tested using the analysis of variance test. To assess the relationship of SAC to several clinical parameters and the LV geometry pattern (normal geometry, concentric remodeling, eccentric LVH, and concentric LVH, in order), we performed univariate and multivariate analyses based on stepwise multiple linear regression. SPSS version 20.0 for Windows (IBM, Armonk, NY, USA) was used for statistical analysis. A value of *p* < 0.05 was considered to indicate statistical significance.

## 3. Results

### 3.1. Clinical and Echocardiographic Characteristics

The mean age of HT patients was 29.8 ± 6.3 years, and 18 (24%) HT patients were women. The total cholesterol level was higher in HT patients than in normal controls (*p* = 0.032). Body mass index was higher in HT patients than in normal controls (*p* = 0.005). The average office SBP and DBP in HT patients were 151.9 ± 15.9 mmHg and 97.2 ± 12.2 mmHg, respectively (Table 1).

There was no difference in LV dimension and LVEF between HT patients and controls. However, HT patients had thicker LV walls and higher RWT and LVMI than normal controls (*p* < 0.001 for all, Table 1). LV systolic wall stress was also higher in HT patients than in normal controls (*p* < 0.001). The left atrial diameter was larger and other LV diastolic parameters including DT, E/A, and e’ velocity were worse in HT patients than in normal controls (*p* < 0.05 for all). LVGLS was significantly lower in HT patients than in normal controls, whereas LVEF was similar between the 2 groups (*p* < 0.001, Table 1).

### 3.2. Relationship between LV Geometry and Function

HT patients were divided into 4 groups, and 30 patients (40%) had any LV geometric changes. HT patients with concentric LVH had higher RWT and LVMI than other HT patients (*p* < 0.001 for both). The e’ velocity was significantly reduced in patients with concentric LVH (Table 2).

Although LVEF was similar among the 4 groups, LVGLS was significantly lower in patients with concentric LVH than in other HT patients (*p* = 0.03, Table 2).

Moreover, LVGLS was lower (−18.5 ± 2.7% vs. −20.0 ± 2.4%, *p* = 0.008) and e’ velocity tended to be lower in HT patients with normal geometry than in normal controls (9.8 ± 1.5 cm/s vs. 10.7 ± 1.7 cm/s, *p* = 0.08). With the cutoff value with −15.2% of LVGLS (mean value of that of normal controls ± 2 standard deviation), the sensitivity and specificity for the detection of LVH were 70.0% and 86.7%, respectively.

### 3.3. Serum Aldosterone and Its Relationship to Left Ventricular Geometry, Function, and Fibrosis

SAC was significantly higher in patients with any LV geometric changes than in patients with normal geometry, and it was the highest in patients with concentric LVH (*p* < 0.001, Table 2). ARR was also higher in patients with concentric LVH than in patients with normal geometry; however, there was a marginal difference in patients with any LV geometric changes (*p* = 0.08). In contrast, PRA was not different among the 4 groups (*p* = 0.48).

**Figure 1:**
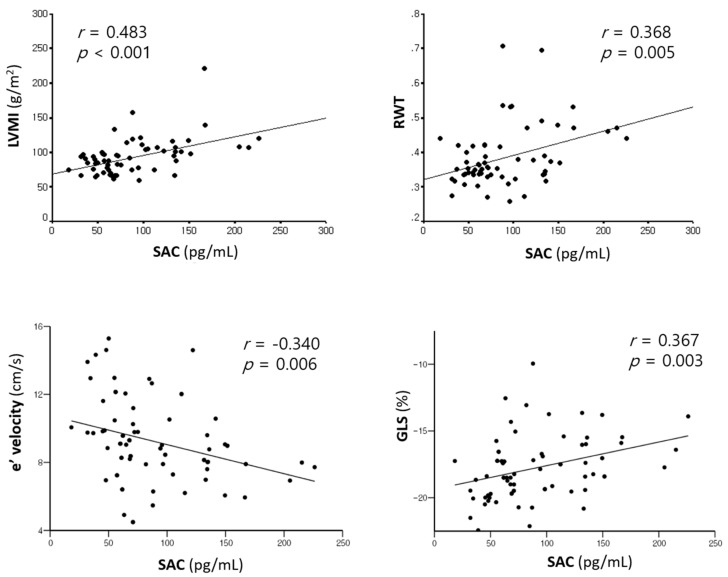
Relationship of SAC to LVMI, RWT, e’ velocity, and LVGLS. LVMI, left ventricular mass index; RWT, relative wall thickness; LVGLS, left ventricular global longitudinal strain; SAC, serum aldosterone concentration.

SAC was positively correlated with LVMI (r = 0.483, *p* < 0.001) and RWT (r = 0.368, *p* = 0.005), and negatively related to LVGLS (r = −0.367, *p* = 0.003) and e’ velocity (r = −0.340, *p* = 0.006) (Table 3, Figure 1). SAC was also significantly related to PIIINP (r = 0.603, *p* = 0.013).

PIIINP was higher in HT patients with concentric LVH than in those with other LV geometry(*p* = 0.026, Table 2), and it was related to LVMI (r = 0.492, *p* = 0.043).

The LVGLS for the systolic function was related to BP parameters and change of the LV geometry pattern to concentric LVH. SAC was most related to LV geometry and average DBP (Table 3). LV diastolic function according to e’ velocity was related to age, average DBP, LV geometry, SAC, ARR, and PIIINP, and was most related to change in LV geometry pattern to concentric LVH and to PIIINP (Table 3).

Change of the LV geometric pattern to concentric LVH was independently related to SAC beyond age and BP (*p* = 0.014, Table 4).

## 4. Discussion

To the best of our knowledge, this is the first study to assess the relationship of aldosterone to LV geometry and function in young adults with never-treated HT. The main findings of this study are as follows: (1) Young HT patients had higher LVMI, higher RWT, and worse LV function than normal controls. LVGLS and e’ velocity were worse in HT patients even with normal geometry than in normal controls. (2) SAC was well correlated with LVMI, RWT, LVGLS, and e’ velocity. (3) SAC was related to PIIINP, which was higher in concentric LVH than in other LV geometries. (4) LVGLS was most related to LV geometry and DBP, whereas e’ velocity was most related to PIIINP and LV geometry. (5) LV geometric change to concentric LVH was most related to SAC beyond age and BP.

The development of LVH is influenced by various neurohumoral factors, and the RAAS is strongly involved. Many studies have suggested that an elevated aldosterone level can contribute to LVH beyond the effects of the BP-related hemodynamic load on such a hypertrophy.

Excess plasma aldosterone in patients with primary aldosteronism is frequently associated with an increase in LV mass beyond levels needed to compensate for the BP-related hemodynamic load in this condition [19,20]. In addition, the existence of a relationship between plasma aldosterone and LV mass has been consistently reported in patients with primary HT [21,22,23,24] and several studies on the use of mineralocorticoid receptor antagonists have indicated that aldosterone has a certain role in LVH and diastolic dysfunction in primary HT, with important implications for the treatment of these conditions [25,26].

Age-related changes in the RAAS in normal humans are well recognized. PRA and SAC decrease with aging, and they are the highest in newborns and the lowest in elderly persons.

In addition, the RAAS has an important role in the BP increase induced by increased peripheral vascular resistance and early structural damage in younger people with a hypertensive phenotype. However, the superimposition of a disease process or the prescription of a drug to induce a change in electrolyte balance, inhibiting renin release, or angiotensin II production could precipitate hyporeninemic hypoaldosteronism [27].

Although some investigations have described the relationship between LV geometry and aldosterone in HT patients who are already receiving treatment [28,29], there have been controversies on the impact of aldosterone on LV function, particularly diastolic function [28,30,31]. With aging, the LV structure undergoes concentric remodeling, and LV diastolic function starts to become impaired. Most studies on aldosterone have been conducted in patients with advanced age, and the impact of aldosterone might be attenuated.

In this study, we attempted to exclude confounding factors affecting the RAAS, such as aging, antihypertensive medication, and renal dysfunction. Finally, a narrow spectrum of young patients was enrolled.

Inappropriate activation of the RAAS and a lack of ability to lower aldosterone levels in response to the high salt intake in modern diets are considered maladaptations of this regulatory system.

Several reports have revealed that aldosterone promotes LV remodeling and interstitial fibrosis via direct effects on the cardiac extracellular matrix [32], changes in cardiac loading conditions through its effects on renal sodium and water retention [33], and increase in arterial stiffness [34].

Myocardial fibrosis and the associated alterations of the extracellular matrix are the major determinants of a hypertensive heart [35], and interstitial and perivascular fibrosis is likely to primarily affect the subendocardium. Longitudinal fibers, as a consequence of their prominent subendocardial location, are more vulnerable to fibrosis and hemodynamic overload. In our study, PIIINP, as one of the myocardial fibrosis markers, was significantly increased in patients with concentric LVH. Aldosterone has been shown to increase the extracellular matrix and collagen deposition in the myocardium by enhancing the expression of cardiac collagen type I and III genes [36], and our study showed a good relationship between SAC and PIIINP. Moreover, such interstitial fibrosis in cardiac tissues may influence not only LV geometry but also LV diastolic function. Studies conducted in patients with primary aldosteronism have shown abnormal LV diastolic properties in association with LVH [37,38]. However, the results of studies that assessed the possible contribution of plasma aldosterone levels to LV diastolic dysfunction in patients with HT were inconsistent [28,30]. Our results showed that the e’ velocity as an LV diastolic parameter was associated with age, BP, LVH, SAC, and PIIINP. In multivariate analysis, increased PIIINP was one of the independent factors for reduced e’ velocity as an LV diastolic impairment. Therefore, aldosterone seems to be related to LV diastolic impairment through myocardial fibrosis, which might occur in the progressed stage of HT and LVH.

SAC was related to an increase in LV mass, concentric remodeling, and myocardial fibrosis. In addition, SAC was related to both systolic and diastolic impairment. In multivariate analysis, change in LV geometry pattern to concentric LVH was most related to SAC. Therefore, it can be assumed that SAC is more related to LV structural remodeling than functional remodeling, and functional remodeling may be due to multiple factors, including age, BP, and myocardial fibrosis. Moreover, the functional impairment might not yet be highly progressed because of the young age of the patients.

Two-dimensional speckle-tracking imaging is a simple and noninvasive echocardiographic technique that can detect subtle changes in myocardial function in HT patients with preserved LVEF [4,39,40,41]. There were significant differences in LVMI, RWT, and LVGLS between normal controls and HT patients despite their young age. Moreover, LVGLS was lower in HT patients with normal LV geometry than in normal controls. Pressure overload-related LVH and myocardial fibrosis frequently appear in the subendocardial layer, leading to the impairment of longitudinal LV function. A decrease in LV longitudinal function can be the early change in response to increased afterload [4,39,40,41].

### 4.1. Study Limitation

The major limitation of our study was the relatively small number of HT patients, even if the accurate selection of never-treated young HT patients could justify this small number. We consecutively enrolled young HT patients who were referred to a tertiary hospital. Moreover, to minimize other confounding factors, we excluded patients who had significant comorbidities, such as diabetes mellitus, renal insufficiency, and already decreased LVEF. Only 18 of the 75 HT patients were women, consistent with the reported low prevalence of HT in young women, and we did not find any sex-related differences. Second, PIIINP was not obtained in all study subjects, and even in HT patients with normal geometry, PIIINP may be higher than that of normal controls. Third, other biological and neurohumoral factors that could affect the results were not evaluated. Lastly, a long-term follow-up with use of mineralocorticoid receptor antagonists is required to evaluate the change in LV geometry and function.

### 4.2. Clinical Implication

HT is an established risk factor for cardiovascular diseases, especially premature atherosclerosis and heart failure. Therefore, early and accurate identification of patients with myocardial dysfunction but without symptoms is considered the main starting point for the development of effective primary prevention plans. However, young HT patients tend to ignore the importance of BP management. Most young HT patients undergo a screening test to evaluate the possibility of secondary HT, including aldosterone and renin tests. This study indicates that SAC can be a good biomarker for subclinical LV remodeling and dysfunction, although the values are within the normal range. Detecting signs of myocardial damage by using 2D speckle-tracking imaging can be useful in evaluating patients in the early stage. In addition, more advanced progression to concentric LVH and fibrosis induced by HT and SAC results in reduced e’ velocity as an LV diastolic impairment. Therefore, more aggressive management is considered for preventing further damage in HT patients with elevated SAC and LV geometric change, even in young patients.

## 5. Conclusions

High serum aldosterone level was related to an increase in LV mass and concentric remodeling, as well as to a decrease in both LV systolic and diastolic function. Serum aldosterone was also associated with myocardial fibrosis. Our findings may indicate that in young patients with never-treated primary HT, aldosterone significantly contributes to changes in LV geometry and functional impairment through its pro-hypertrophic and myocardial fibrosis effects.

## Figures and Tables

**Table 1:** Characteristics of normal controls and young never-treated hypertensive patients.

	Normal Controls, (*n* = 45)	Young Never-Treated HT Patients (*n* = 75)	*p*-Value
**Men/Women** (*n*)	30/15	57/18	0.29
**Age** (years)	28.9 ± 5.1	29.8 ± 6.3	0.42
**Body mass index** (kg/m^2^)	23.4 ± 3.0	25.5 ± 4.2	0.005
**Heart rate** (/min)	70 ± 11	72 ± 9	0.26
**SBP** (mmHg)	120.1 ± 10.9	151.5 ± 15.9	<0.001
**DBP** (mmHg)	75.3 ± 9.3	97.2 ± 12.2	<0.001
**Smoking** (*n*, %)	8 (16%)	10 (20%)	0.80
**Creatinine** (mg/dL)	0.90 ± 0.25	0.97 ± 0.16	0.15
**Total Cholesterol** (mg/dL)	168.4 ± 34.6	182.5 ± 35.0	0.032
**Triglycerides** (mg/dL)	121.9 ± 55.4	142.8 ± 74.9	0.22
**Glucose** (mg/dL)	91.8 ± 7.7	95.6 ± 10.9	0.12
**Uric acid** (mg/dL)	5.5 ± 1.8	6.1 ± 1.6	0.24
**eGFR**, mL/min/ 1.73 m^2^	105 ± 17	102 ± 22	0.49
**Sodium**, mmol/L	139 ± 3	140 ± 2	0.85
**Potassium**, mmol/L	4.3 ± 0.3	4.1 ± 0.3	0.45
**LVEF** (%)	61.5 ± 6.2	63.1 ± 6.0	0.88
**LVMI** (g/m^2^)	80.0 ± 12.7	101.5 ± 16.3	<0.001
**RWT**	0.33 ± 0.04	0.40 ± 0.09	<0.001
**LV systolic wall stress** (10^3^ dynes/cm^2^)	73.8 ± 14.9	87.4 ± 19.9	<0.001
**Left atrial diameter** (mm)	30.8 ± 4.1	32.8 ± 4.5	0.02
**E-velocity** (cm/s)	74.3 ± 16.4	71.7 ± 18.0	0.52
**DT of E** (ms)	178.3 ± 31.8	195.1 ± 49.1	0.04
**E/A**	1.8 ± 0.5	1.6 ± 0.5	0.02
**e’ velocity** (cm/s)	10.7 ± 1.7	9.6 ± 2.7	0.02
**E/e’**	5.7 ± 1.0	7.8 ± 2.6	0.10
**LVGLS** (%)	−20.0 ± 2.4	−17.9 ± 2.9	<0.001

Abbreviations: HT, hypertensive, SBP, systolic blood pressure; DBP, diastolic blood pressure; eGFR, estimated glomerular filtration rate; LV, left ventricle; LVEF, left ventricular ejection fraction; RWT, relative wall thickness; LVMI, left ventricular mass index; LVGLS, left ventricular global longitudinal strain; E, early diastolic mitral inflow velocity; DT, deceleration time; E/A, late diastolic mitral inflow velocity; e’, early diastolic mitral annular velocity; E/e’, the ratio of early diastolic mitral inflow velocity to early diastolic mitral annular velocity; LVGLS, left ventricular global longitudinal strain.

**Table 2:** Comparison of clinical and echocardiographic parameters according to left ventricular (LV) geometry in young never-treated hypertensive patients.

	Normal Geometry (*n* = 45)	Concentric Remodeling (*n* = 5)	Eccentric LVH (*n* = 10)	Concentric LVH (*n* = 15)	*p* *-Value*
**Age** (years)	29.8 ± 6.2	30.5 ± 10.8	31.4 ± 5.5	30.9 ± 10.9	0.43
**LVEF** (%)	61.9 ± 5.4	62.5 ± 6.3	61.7 ± 9.2	63.5 ± 7.3	0.79
**RWT**	0.35 ± 0.04	0.43 ± 0.07	0.36 ± 0.03	0.53 ± 0.07	<0.001
**LVMI** (g/m^2^)	80.3 ± 12.2	91.7 ± 10.3	115.0 ± 12.1	131.7 ± 15.3	<0.001
**DT of E** (ms)	192 ± 51	197 ± 50	200 ± 52	203 ± 48	0.79
**e’ velocity** (cm/s)	9.8 ± 1.5†	9.1 ± 2.2	8.8 ± 2.4	7.4 ± 1.1	0.01
**LVGLS** (%)	−18.6 ± 1.8*	−17.9 ± 2.9	−16.8 ± 2.7	−15.9 ± 2.4	0.002
**Sodium**, mmol/L	140 ± 2	139 ± 3	140 ± 3	140 ± 3	0.95
**24 h urinary sodium**, mEq/d	152 ± 86	154 ± 68	148 ± 79	145 ± 51	0.10
**eGFR**, mL/min/ 1.73 m^2^	101 ± 23	102 ± 23	99 ± 25	100 ± 22	0.55
**SAC** (pg/mL)	64.2 ± 26.9	113.5 ± 22.9	115.9 ± 25.4	153.5 ± 21.9	<0.001
PRA (ng/mL/hr)	2.68 ± 2.43	2.83 ±1.91	2.79 ± 1.55	3.62 ±1.41	0.48
**ARR**	34.0 ± 19.5	40.3 ± 20.4	46.0 ± 20.1	51.6 ± 24.4†	0.08
**PIIINP** (U/mL)	0.47 ± 0.13	0.53 ± 0.07	0.50 ± 0.06	0.78 ± 0.27	0.026

*p < 0.05, Normal controls vs. Normal geometry; † *p* = 0.08, Normal controls vs. Normal geometry. Abbreviations: LVEF, left ventricular ejection fraction; RWT, relative wall thickness; LVMI, left ventricular mass index; DT, deceleration time; e’, early diastolic mitral annular velocity; LVGLS, left ventricular global longitudinal strain; SAC, serum aldosterone concentration; PRA, plasma renin activity; ARR, aldosterone-to-renin ratio; PIIINP, procollagen type III amino-terminal peptide. *n* = 45 (Normal geometry = 27, Concentric Remodeling = 3, Eccentric LVH = 6, Concentric LVH = 9).

**Table 3:** Correlation of left ventricular global longitudinal strain (LVGLS) and e’ velocity with various parameters.

	LVGLS					e’ Velocity				
	Univariate		Multivariate			Univariate		Multivariate		
	*r*	*p*-Value	β	95% CI	*p*-Value	*r*	*p*-Value	β	95% CI	*p*-Value
**Age**	0.061	0.657				−0.400	0.002			
**Average SBP**	−0.188	0.182				−0.141	0.306			
**Average DBP**	−0.368	0.007	−0.328	−25.483–−4.300	0.010	−0.351	0.009			
**LV geometry**	0.439	<0.001	−0.253	−16.511–−0.006	0.047	−0.485	<0.001	−0.386	−1.192–−0.001	0.050
**SAC**	−0.288	0.033				−0.413	0.001			
**ARR**	0.085	0.536				−0.274	0.040			
**PIIINP**	−0.341	0.130				−0.593	0.005	−0.566	−1.149–−0.019	0.024

Abbreviations: SAC, serum aldosterone concentration; BMI, body mass index; SBP, systolic blood pressure; DBP, diastolic blood pressure; LV geometry, normal geometry, concentric remodeling, eccentric LVH, and concentric LVH in order; e’, early diastolic mitral annular velocity; LVGLS, left ventricular global longitudinal strain; SAC, serum aldosterone concentration; ARR, aldosterone-to-renin ratio; PIIINP, procollagen type III amino-terminal peptide. *n* = 45.

**Table 4:** Correlation of LV geometry with various parameters.

	Univariate		Multivariate		
	*r*	*p*-Value	β	95% CI	*p*-Value
**Age**	0.033	0.808			
**Average SBP**	0.258	0.057			
**Average DBP**	0.372	0.014	0.277	0.000–48.021	0.050
**SAC**	0.706	<0.001	0.709	21.678–89.143	0.019
**ARR**	0.243	0.046			
**PIIINP**	0.526	0.014			

Abbreviations: SAC, serum aldosterone concentration; BMI, body mass index; SBP, systolic blood pressure; DBP, diastolic blood pressure; LV geometry, normal geometry, concentric remodeling, eccentric LVH, and concentric LVH in order; SAC, serum aldosterone concentration; ARR, aldosterone-to-renin ratio PIIINP, procollagen type III amino-terminal peptide. *n* = 45.

## Supplementary Materials

The following are available online at https://www.mdpi.com/2077-0383/8/7/1045/s1, Supplementary File S1: Measurement of left ventricular global longitudinal strain (LVGLS).
