# Supplementary material for: Serum Aldosterone Is Related to Left Ventricular Geometry and Function in Young Adults with Never-Treated Primary Hypertension
Source: J Clin Med. 2019 Jul 17;8(7):1045. doi: 10.3390/jcm8071045 (PMC6679090; doi:10.3390/jcm8071045)
Supplement: Supplementary file 1 [file jcm-08-01045-s001.pdf]

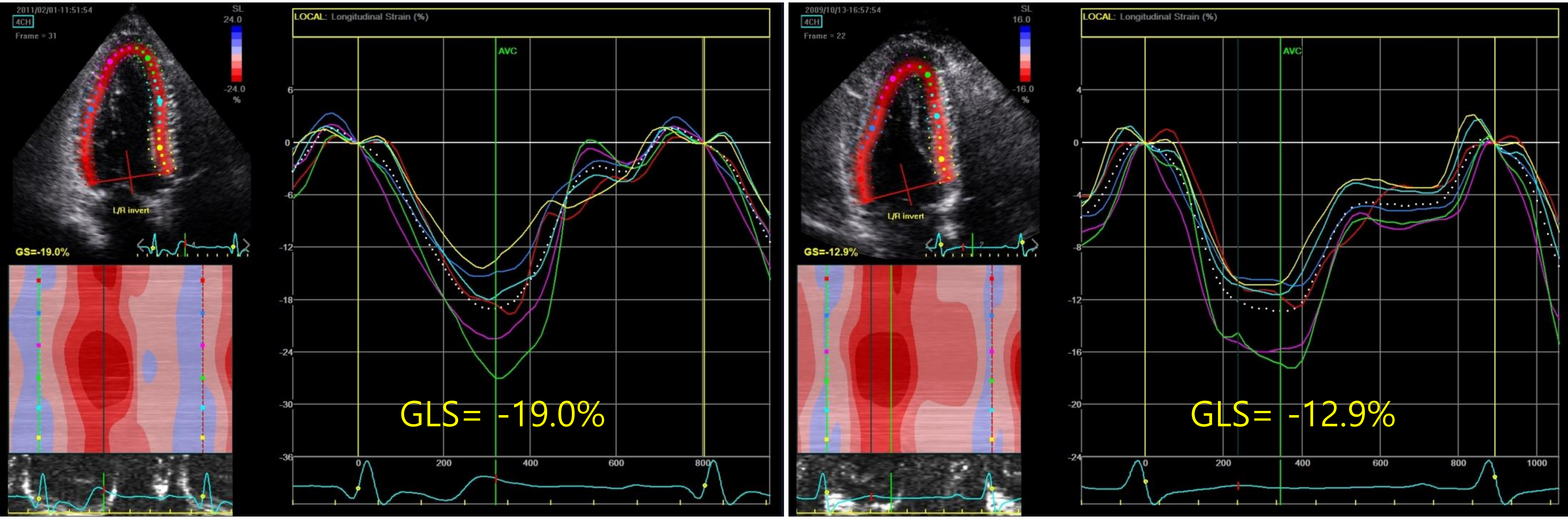

Measurement of left ventricular global longitudinal strain (LVGLS). From apical 4 chamber images, LVGLS (white dot curve) was -19.0% in normal control (left), but was reduced by -12.9% in hypertensive patients with left ventricular hypertrophy (right).
